# Research Progress on Additively Manufactured Diamond Tools

**DOI:** 10.3390/ma18245540

**Published:** 2025-12-10

**Authors:** Chenchen Tian, Chi Chen, Yi Wan, Xuekun Li

**Affiliations:** 1 School of Mechanical Engineering, Shandong University, Jinan 250061, China; 2 Key Laboratory of High Efficiency and Clean Mechanical Manufacture of Ministry of Education, Shandong University, Jinan 250061, China; 3 Department of Mechanical Engineering, Tsinghua University, Beijing 100084, China

**Keywords:** diamond tools, additive manufacturing, 3D printing, grinding tools, cutting tools, drilling tools

## Abstract

With their exceptional hardness and wear resistance, diamond tools hold an irreplaceable position in critical fields such as precision machining, geological exploration, and construction engineering. However, traditional manufacturing processes like powder metallurgy still face numerous limitations in terms of structural design optimization, the controllability of diamond particle distribution, and the shortening of production cycles. In recent years, additive manufacturing has emerged as a disruptive technology that precisely constructs three-dimensional structures in a layer-by-layer manner, offering new possibilities for the customized design and functionally integrated manufacturing of high-performance and complex-structured diamond tools. This paper systematically reviews the recent research progress on the additive manufacturing of diamond tools. It focuses on summarizing the fabrication characteristics and performance of metal-bonded diamond tools, resin-bonded diamond tools, and ceramic-bond diamond tools prepared by different additive manufacturing processes. On this basis, the paper further discusses the key technical challenges and future development directions in this field, with the aim of providing a theoretical reference and technical guidance for the design optimization and engineering applications of additively manufactured diamond tools.

## 1. Introduction

As a representative of superhard materials, diamond has unique physical and chemical properties that are irreplaceable in the field of high-end manufacturing. Diamond tools can be distinguished according to the type of binder, mainly including metal-bonded tools, resin-bonded tools, and ceramic-bonded tools. When classified by core function, diamond tools include grinding tools, drilling tools, cutting tools. and dressing tools. Due to their exceptional strength, hardness, and wear resistance, diamond grinding wheels, drill bits, saw blades, and cutting tools are widely used in the processing of hard and brittle materials such as ceramics, glass, semiconductor materials, and stones [1,2,3].

The traditional preparation methods for diamond tools mainly include hot-pressing sintering, electroplating, and brazing. Hot-pressing sintering has long been the dominant method in diamond tool manufacturing. By applying simultaneous pressure and heat, it significantly accelerates the contact diffusion between powder particles, ultimately yielding products with fine grains and high density [4]. However, this process exhibits limited capability in forming special-shaped, ultra-thin, or miniaturized tools and porous structures. During cutting or grinding operations, its poor heat dissipation performance makes burn damage prone to occurring. Consequently, hot-pressing sintering struggles to meet the manufacturing demands for diamond tools with complex geometries [5]. The electroplating process for manufacturing diamond tools exhibits characteristics of high machining accuracy, good surface quality, and low processing costs [6]. However, due to the characteristics of the electroplating process, the optional types of matrix metals are limited to a few metals such as Ni and Co and their alloys, which are far less than the metal selection range of the powder metallurgy process. Electroplating is mechanically bonded, with defects such as insufficient adhesion between the coating and the substrate, internal stress accumulation, and an interface gap, which can easily cause local peeling of the coating or diamond particles falling off, significantly reducing the tool life and processing efficiency [7]. The brazing process for manufacturing diamond tools can form a robust metallurgical bond between the diamond, brazing alloy and matrix, enhance the holding force and protrusion height of diamond, and improve the self-sharpening and cutting performance. The high brazing temperature during the manufacturing process of brazed diamond tools can easily cause thermal damage such as graphitization, lattice microcracks, and chemical erosion on the diamond surface, resulting in a decrease in diamond abrasive strength. At the same time, most of the existing single-layer manufacturing processes reduce the service stability and service life of tools [8,9].

With the development of diamond tools, their shape has become increasingly complex, and the structure has become more and more refined. At the same time, the demanding performance has been continuously improved. The traditional diamond manufacturing process has limitations in the manufacturing of diamond tools with complex structures, making it difficult to meet the growing demand. Therefore, it is urgent to explore new manufacturing technologies for diamond tools. Compared with the traditional diamond tool manufacturing process, additively manufactured technology simplifies the three-dimensional construction to a two-dimensional plane construction by building a digital model and using materials such as powder, wire, liquid, and sheet to build a solid layer by layer, greatly expanding the degree of design freedom. This technology has the advantages of high precision, high density, printing complex structures (special-shaped, ultra-thin, porous, lattice, internal flow channel microstructure, etc.) and forming metallurgical bonding under the action of a laser to enhance the control of matrix and diamond particles. It is widely used in the manufacture of products in aerospace, medical, automotive, and other fields. With the help of selective laser melting, selective laser sintering, stereolithography, and other technologies, scholars have explored the manufacturing of a variety of diamond tools.

**Figure 1:**
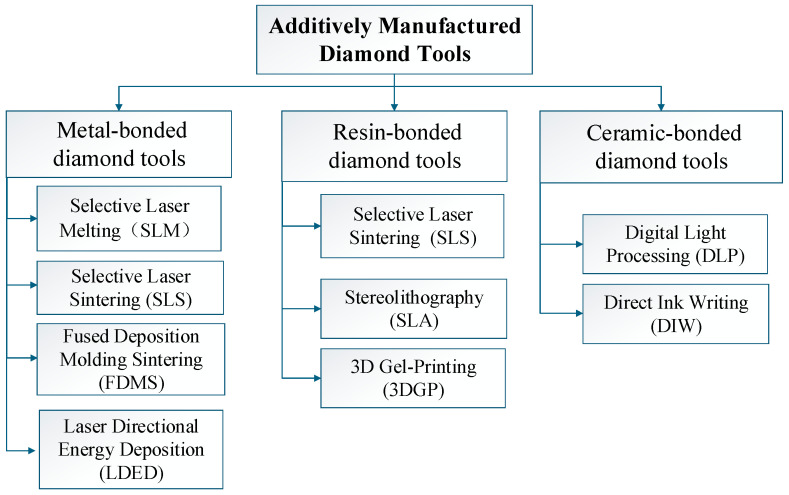
Classification of additively manufactured diamond tools.

This paper systematically summarizes the research progress in the field of additively manufactured diamond tools in recent years, focusing on the technical system of metal-bonded, resin-bonded, and ceramic-bonded additively manufactured diamond tools (as shown in Figure 1), summarizes the laser power range and wavelength corresponding to different processes (as shown in Table 1), and analyzes the common challenges and development trends in this field. The purpose is to provide a theoretical and practical reference for the digital manufacturing technology and engineering application of high-performance and personalized diamond tools.

## 2. Additively Manufactured Metal-Bonded Diamond Tools

Metal-bonded diamond tools have the advantages of a high bonding strength, good wear resistance, high cutting efficiency, and long service life, but their self-sharpening property is poor, grinding efficiency is low, and they easily burn and clog if used improperly. Compared with diamond tools bonded on a ceramic matrix, metal bond types often perform poorly in terms of porosity. In the manufacturing process, the process range of forming pores by a metal bond is more limited, and it is difficult to accurately control and adjust that range. Although pore-forming agents can increase porosity, chip space, and the heat dissipation capacity to a certain extent, the pores generated by pore-forming agents have such phenomena as a shape, size, uneven distribution, and weakened bearing strength. Therefore, additively manufactured technology is introduced, which can form a three-dimensional pore structure with a controllable morphology and interconnection. As early as 2004, the possibility of laser additively manufactured diamond tools was verified [10]. At present, the additively manufactured technologies used to manufacture metal-bonded diamond tools are mainly selective laser melting (SLM), selective laser sintering (SLS), fused deposition molding sintering (FDMS), and laser directional energy deposition (LDED).

### 2.1. Fabrication of Metal-Bonded Diamond Tools by SLM

**Figure 2:**
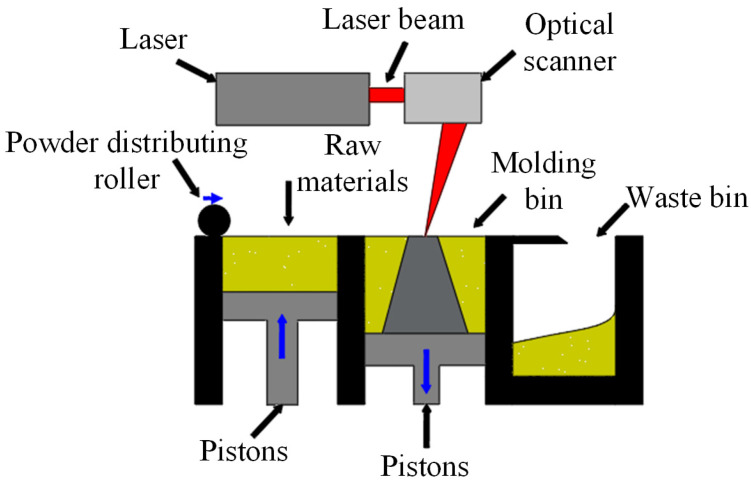
Basic principle of SLM. Adopted with permission from Chen et al. [17].

SLM is an advanced additive processing technology for free-form surface manufacturing. Its working principle is shown in Figure 2. The process uses a high-energy laser beam as an input heat source, alloy powder as a molding material. Modeling was performed using SOLIDWORKS 2020, and slicing was conducted using Materialise Magics 23.0. In operation, the 3D model is decomposed into several 2D sections, and the process path is accurately designed according to these sections. A continuous-wave fiber laser (wavelength 1070 nm, power 100–1000 W, repetition rate 1–5 kHz) passes through the optical deflection system (such as a galvanometer) to scan each layer section and melt the metal powders in a protective atmosphere, thereby constructing a complete three-dimensional structure layer by layer. Compared with traditional manufacturing methods, SLM technology has significant advantages, including a shorter R&D cycle, higher design freedom and accuracy, rapid prototyping of complex geometry, and higher utilization of alloy powder. These characteristics mean SLM technology has wide application value in many fields [11,12,13,14,15,16].

The core of metal-bonded diamond tool research is to optimize and improve the performance of metal-bonded diamond material. Researchers have proposed a variety of solutions to improve material properties from the aspects of printing parameter optimization, alloy composition regulation, model simulation, and so on.

Su et al. [18] fabricated the composite of diamond particles and AlSi10Mg through the SLM process, but found that the metallurgical bonding effect was poor. Wang et al. [19] tried to prepare an AlSi10Mg/diamond composite by pretreatment with titanium plating and SLM technology, but there were still pores at the interface. Tian et al. [20,21] determined the optimal parameters of an AlSi10Mg/diamond composite fabricated by SLM through an orthogonal experiment and proved the feasibility of using SLM to prepare a metal-bonded diamond grinding wheel.

Zhang et al. [22] fabricated dense Cu/diamond composites through SLM technology, achieving good interface bonding. Gao et al. [23] designed a W/Co double-layer coating for diamond particles. Specimens were fabricated by SLM, and it was found that diamond and matrix metallurgical bonding were good. Spierings et al. [24] used SLM technology to prepare a Cu-Sn-Ti-Zr/Ni-plated diamond active filler metal composite, and diamond particles were closely combined with the matrix. Rommel et al. [25] used SLM technology to manufacture Ni-bonded diamond tools, and the diamond was firmly embedded in the matrix.

Zhang et al. [26] found through the discrete element method model that irregular diamond particles perform poorly during the powder laying process, in the process of powder spreading, resulting in segregation. Increasing the thickness of the powder layer and decreasing the powder spreading speed can improve the quality of the powder bed and then improve the SLM printing quality. The computational fluid dynamics model established by Xu et al. [27] shows that the CuSn20 bonded diamond composite fabricated by SLM can form a continuous molten pool without diamond falling off at 200 W laser power. Gan et al. [28] determined the SLM process parameters by simulating the temperature field of the molten pool and found that the wear resistance of the specimens fabricated by SLM was better than that of the hot-pressed sintered specimens. Zhou et al. [29] simulated the laser additively manufactured process of Ni-Cr metal-bonded diamond tools and pointed out that the thickness of the powder layer has a great impact on the energy absorption rate, and diamond affects the temperature and shape of the molten pool. During multi-channel scanning, it is necessary to adjust the scanning spacing and laser energy density to reduce the bath temperature and remelting area and reduce the thermal damage of the diamond.

SLM technology is especially suitable for manufacturing diamond tools with complex structures such as grids and lattices. This technology not only greatly improves the bearing capacity of the tool but also significantly improves the working efficiency and cutting and grinding performance. In addition, SLM technology effectively solves the problem of a narrow chip removal channel caused by the dense structure in the process of traditional manufacturing of metal-bonded diamond tools [30].

Peng et al. [31] fabricated 60° V-shaped, octahedral, square, and spherical pore structures using SLM technology with FeCoCrNi high-entropy alloy powder as a metal bond and diamond coated with inner-layer Ti and outer-layer Ni as abrasive particles. The results show that the porous specimens fabricated by diamond abrasives with a porosity of 30%~50% have no defects, good structural integrity, and good connectivity of pore units. The optimal square pore structure model with a porosity of 50% has a uniform stress distribution and the lowest stress concentration, which is attributed to the frame support and uniform wall thickness of the pore unit, showing better mechanical properties, good sharpness, and self-sharpness. Han et al. [32] successfully fabricated porous diamond tools with controllable porosity (30–70%) by using a TPMS honeycomb Gyroid for filling holes through SLM technology. The compression test shows that with the increase in porosity, the elastic modulus and compressive strength of the specimen gradually decrease and the failure mechanism of the specimen fabricated by SLM changes from diagonal shear failure to layer-by-layer fracture. For the specimens with low porosity, the stress distribution is more uniform, while for the specimens with high porosity, the stress distribution is concentrated in the thin column. Compared with solid grinding tools, porous grinding tools have an excellent grinding performance and can process higher-quality workpieces.

**Figure 3:**
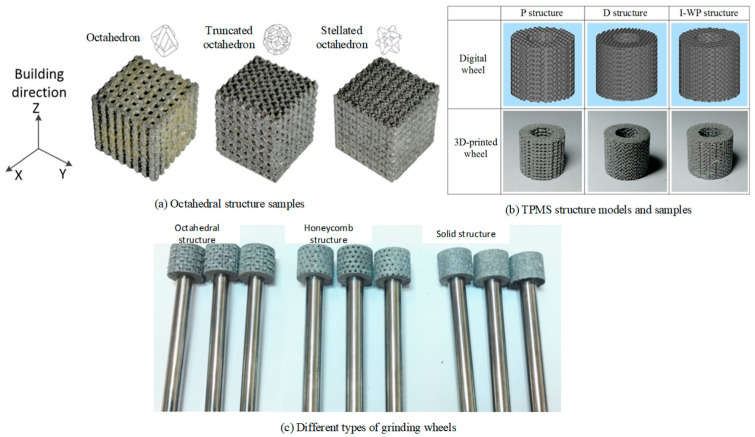
Metal-bonded porous diamond tool printed by SLM. Adopted with permission from Tian and Rahmani et al. [33,36,37].

Tian et al. [33,34,35,36,37,38] designed and fabricated a variety of porous grinding wheel structures based on an AlSi10Mg/diamond composite using the SLM process. Their work mainly includes three parts: firstly, three kinds of porous structure grinding wheels, octahedron, truncated octahedron, and stellated octahedron, are designed (see Figure 3a); secondly, grinding wheels with octahedral, honeycomb, and solid structures were fabricated comparatively (see Figure 3c); finally, based on the theory of a Three-Period Minimal Surface (TPMS), the porous grinding wheel structures of Schwarz P, Schwarz D, and Schoen I-WP are constructed (see Figure 3b). The research shows that the porosity can reach at least 30% in the study of the structure of truncated octahedral and stellated octahedral grinding wheels. The two structures show different performance characteristics: the truncated octahedron structure usually has higher porosity, but its compressive strength is relatively low; in contrast, the stellated octahedron structure shows higher compressive strength although its porosity is lower. In the study of the effect of porosity on the mechanical properties of a grinding wheel, a honeycomb structure grinding wheel with porosity in the range of 30~70% was fabricated. It was found that the increase in porosity usually led to a decrease in compressive strength. In addition, when comparing the grinding performance of octahedral, honeycomb, and solid-structure grinding wheels, the surface roughness and surface hardness are used as analysis indices. The experimental results show that the surface roughness of the grinding wheel formed by SLM technology is significantly less than that of the electroplated grinding wheel, which shows the advantages of SLM technology in the preparation of a high-surface-quality grinding wheel. In the research on the design and preparation of three kinds of micro-curved porous grinding wheels (Schwarz P, Schwarz D, and Schoen I-WP), based on TPMS, the pore structure of the grinding wheel was designed through the implicit function equation of TPMS and the pore morphology was accurately controlled by the constraint of pore connectivity and the parameter control equation. The experimental results show that the grinding performance of the SLM wheel is significantly better than that of the electroplated wheel in the grinding process and its grinding force and specific grinding energy are significantly reduced. In addition, this kind of grinding wheel has an excellent dressing and self-sharpening ability, highly controllable pore structure and porosity, and good comprehensive grinding performance. The connected pore structure further enhances the heat dissipation capacity and increases the chip space.

Rahmani et al. [39,40,41] adopted a composite preparation technology combining SLM and Spark Plasma Sintering (SPS) in order to improve the wear resistance of equipment in harsh application scenarios such as mining. Firstly, using SLM technology, using 316L stainless steel or Ti6Al4V alloy as materials, the diamond honeycomb lattice and functionally graded honeycomb lattice were fabricated. Then, on the basis of this structure, Ni-plated diamond particles were added and sintered by SPS technology. The results show that the uniform distribution of diamond particles in the metal matrix is successfully achieved by this technology. Based on the three-stage FGL design, the material presents an optimized performance distribution in structure: more metal is concentrated in the bottom area, which significantly improves the weldability and ductility of the material; the top area shows higher hardness because it can accommodate more hard materials. The results show that the wear resistance of the composite is further enhanced with the increase in diamond content. In the SPS process, the graphitization tendency of diamond at a high temperature was effectively inhibited by using Ni-coated diamond particles and a rapid sintering strategy. The gradient lattice structure is verified by finite element analysis and the simulation results show that the structure can improve the service performance of the material under the conditions of abrasion and impact load.

### 2.2. Fabrication of Metal-Bonded Diamond Tools by SLS

**Figure 4:**
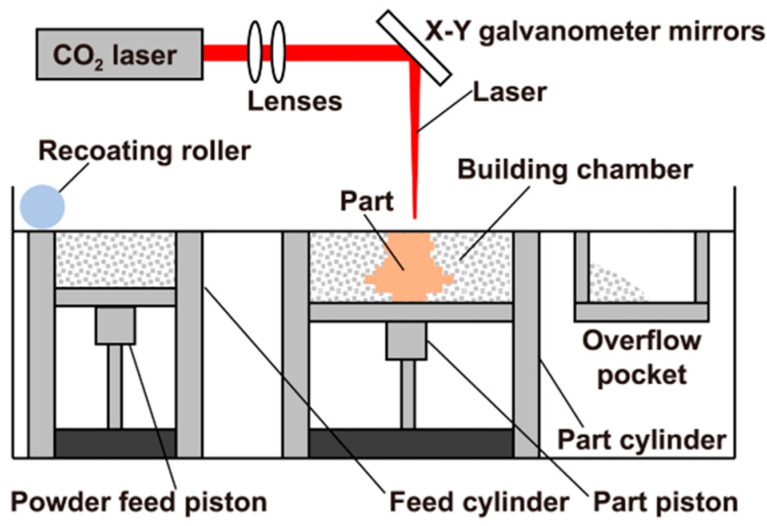
Basic principle of SLS. Adopted with permission from Han et al. [45].

SLS technology uses a laser as the power source to sinter thin layers of powder materials on the printing platform layer by layer. High-energy laser selectively melts powder particles and solidifies them into three-dimensional objects. Specifically, as shown in Figure 4, the laser scans the cross-section of the part according to the digital 3D model and sinters it on the powder surface. After each layer of sintering, the thickness of the powder layer will be reduced and a new layer of material will be covered. This process is repeated until the part is fully formed. The technology has significant advantages, including a low manufacturing cost, short processing time, and high material utilization. In addition, it can print metal or polymer parts with a complex geometric structure directly without a supporting structure. These parts are of high strength and rigidity and are very suitable for mass production [42,43,44,45].

**Figure 5:**
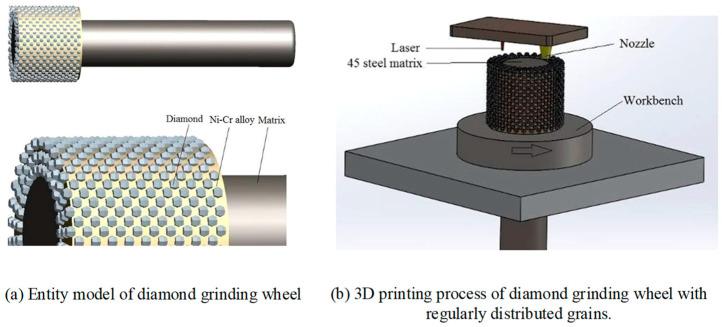
Preparation process and model of diamond grinding wheel with regular arrangement of abrasive grains. Adopted with permission from Yang et al. [48].

In recent years, the use of SLS technology to manufacture metal-bonded diamond tools has also been widely developed. Aiming at the problems of uneven particle distribution and a complex preparation process of diamond grinding wheel, Chen et al. [46] invented a method to prepare a metal-bonded diamond grinding wheel with a regular arrangement of abrasive particles based on SLS technology, which simplifies the preparation process of a special-shaped grinding wheel, reduces the difficulty of building the equipment platform, and makes the operation process simpler and more efficient. The grinding wheel fabricated by this process performs well in the grinding process: the surface quality of the workpiece is high, the grinding efficiency is significantly improved, and the grinding force distribution is more uniform, which improves the processing stability and accuracy. Yang et al. [47,48] successfully fabricated a Ni-Cr alloy/diamond grinding wheel with uniform diamond particles by using SLS technology and controlling process parameters such as the laser power (300–500 W), scanning speed (25–35 mm/min), and beam size (3 mm × 2 mm). The grinding wheel preparation process and solid model are shown in Figure 5a,b. It is found that the energy density has a key effect on the bonding quality of diamond: when the energy density is lower than 342.8 J/mm^2^, the diamond is prone to falling off; when it is higher than 364.2 J/mm^2^, graphitization occurs. However, by controlling the energy density in the range of 342.8~364.2 J/mm^2^, the high-strength combination of diamond and alloy can be achieved and a grinding wheel with a better performance can be obtained. Thanks to the good wettability of Ni-Cr alloy to diamond, the sintered diamond particles are complete in shape and free of cracks and thermal damage. In the heavy-load grinding experiment, the diamond abrasive grains show excellent wear and fracture resistance and do not fall off, and microcracks form a new cutting edge at the tip of the abrasive grains, which is conducive to the whole grinding process.

Impregnated diamond bits have been widely used in drilling engineering fields such as geological exploration, oil and gas exploitation, mining, and construction. Zhang et al. [49,50] designed a diamond-impregnated bit with grid-shaped matrix (DIBGM) fabricated based on SLS technology. CoCrMo alloy and Inconel 718 alloy were selected as matrix materials, respectively, and the mechanical properties of the two were systematically tested. The results show that CoCrMo alloy has a better service life while ensuring drilling efficiency, so it is selected as the final matrix material of DIBGM. In the preparation process, the team used the process parameters of laser power 200–300 W, scanning rate 800–1000 mm/s, and auxiliary powder layer thickness 0.05 mm to print the drill bit working layer. The microstructure observation showed that the diamond in the working layer was evenly distributed without obvious aggregation or defects, which preliminarily proved that the material system had good compatibility with the SLS process. Through rock cutting experiments on DIBGM, it is found that its working layer has a regular morphology and its unique grid structure shows a high cutting performance in hard rock formation. The research also reveals that the thickness of the grid slice is positively correlated with the strength of drillable rock; that is, the thicker the grid slice, the higher the rock strength the bit can deal with. The calculation results show that the specific pressure of the working face at the bottom of the new drill bit is 67% higher than that of the traditional drill bit of the same specification, which means that DIBGM has a better rock pressing and breaking ability. In addition, the grid structure is conducive to the flow of coolant, improves the cooling effect of the diamond, and avoids the early failure caused by thermal damage. In summary, this study proves the feasibility, applicability, and efficiency of a CoCrMo-based grid-impregnated diamond bit from the aspects of material selection, structural design, and actual drilling effect.

**Figure 6:**
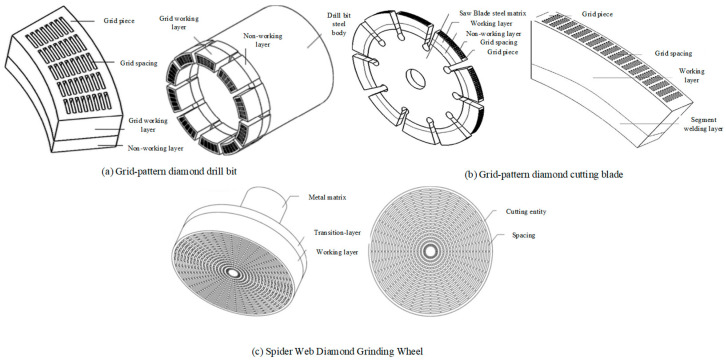
Grid-patterned, spider-web-patterned diamond tools. Adopted with permission from Zhang et al. [51,52,54].

Zhang’s team also used 3D-printing technology to manufacture and perform innovative design and research on a metal-bonded grid diamond saw blade [51], spider mesh diamond grinding wheel [52], and polycrystalline diamond compact [53] with a ripple-shaped gradient layer, and they achieved ideal experimental results (see Figure 6).

### 2.3. Fabrication of Metal-Bonded Diamond Tools by FDMS

**Figure 7:**
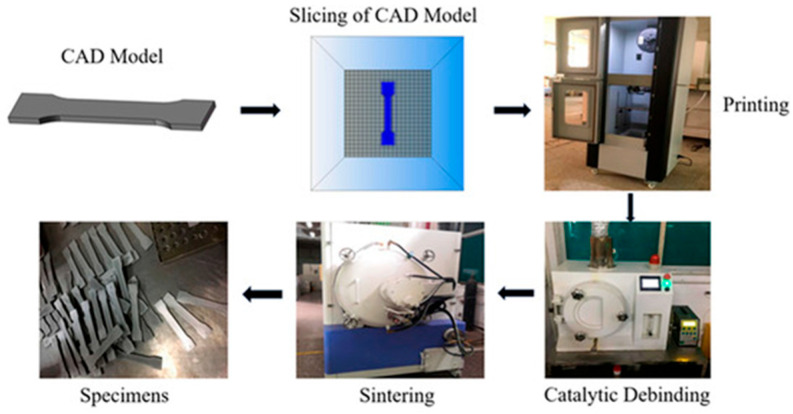
FDMS process flow. Adopted with permission from Wu et al. [59].

FDMS is a kind of additively manufactured method combining fused deposition molding and sintering technology, which is mainly used in the manufacturing of complex structures of high-performance materials such as metals and ceramics. As shown in Figure 7, the process flow is briefly described as follows: first, the required powder is fully mixed and placed in the twin-screw extruder, and then the consumables are obtained by cooling and the fabricated consumables are loaded into the FDM printing equipment. According to the preset model design, the printer extrudes the raw materials through the heating nozzle and stacks them layer by layer to build a three-dimensional model. Subsequently, the printed parts need to undergo degreasing, sintering, and subsequent processing in order to finally obtain the required finished products. The FDMS process has attracted much attention because of its low energy consumption and low cost. Especially when strict strength requirements are not required, such as the production of some functional products, this technology shows significant advantages [55,56,57,58,59].

Zhang et al. [60] successfully fabricated a CoCuSn ultra-thin diamond saw blade by the FDMS process, which showed an excellent alloying degree and compactness, and its CoCuSn/diamond composite also showed good microstructure uniformity. Jin et al. [61] successfully fabricated ultra-thin diamond blades based on Co and CuSn alloy by using the FDMS process and systematically discussed the influence of Co content change on the performance of the blades. The experimental results show that properly increasing the Co content in the matrix is helpful to enhance the overall mechanical properties of the composite and improve the interface adhesion between the matrix and diamond particles. The optimization of this interface combination is directly reflected in the improvement of the macro performance of the blade, including the improvement of the flatness and sharpness of the blade and the enhancement of the overall cutting stability and life.

**Figure 8:**
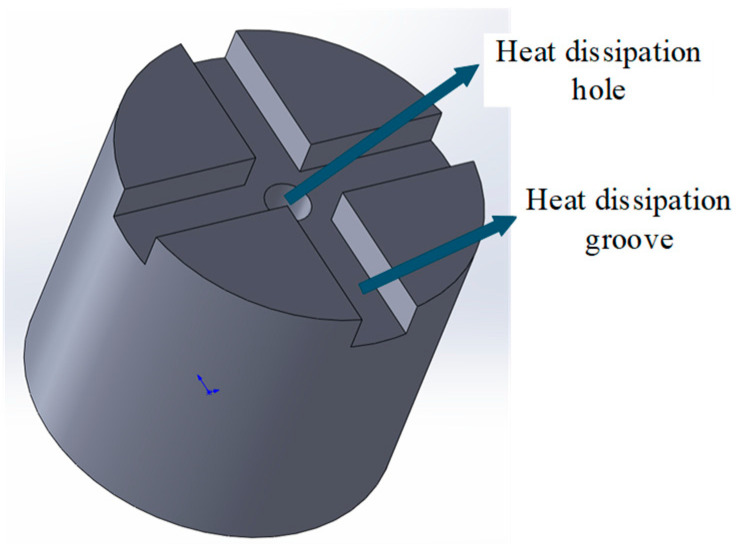
The model of the double-layer diamond grinding head.

Wu et al. [62] proposed a new type of diamond grinding head for slotting cemented carbide. The model of the double-layer diamond grinding head is shown in Figure 8. The grinding head was fabricated by double-nozzle FDMS technology. The structural design of the grinding head includes an outer working layer and an inner non-working layer: the working layer is composed of metal bonds and diamond particles, which are directly involved in grinding; the non-working layer is completely composed of metal bonds, which mainly play the roles of support and force transmission. In order to significantly improve the chip removal, chip holding, cooling, and self-sharpening performance in the slotting process, the research team designed a through-type heat dissipation hole in the center of the grinding head and arranged a crisscross rectangular heat dissipation groove around it. In addition, the effects of diamond concentration and the CO content on the wear resistance of the grinding head were systematically discussed. The experimental results show that increasing the volume concentration of diamond is helpful to improve the overall physical properties of the grinding head, although the density of the material decreases slightly (by 2.47%), its hardness increases significantly (by 10.98%), and the grinding efficiency and wear resistance are enhanced. Co shows good wettability to diamond particles in the matrix, which effectively improves the bonding strength and grinding performance; however, when the CO content exceeds 12%, the cutting performance of the grinding head will decline.

### 2.4. Fabrication of Metal-Bonded Diamond Tools by LDED

**Figure 9:**
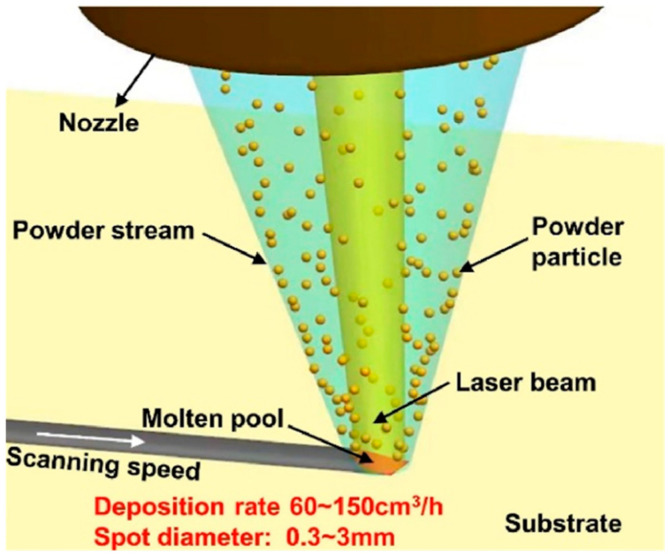
Basic principle of LDED. Adopted with permission from Piscopo et al. [65].

LDED technology integrates the core principles of laser cladding and the welding process. As shown in Figure 9, the technology forms a molten pool on the surface of the substrate through a laser and synchronously sends metal powder or wire into the molten pool area with the help of coaxial or paraxial nozzles. The molten pool is formed by the laser energy melting the substrate surface layer and the transported metal material, and then the metallurgical bonding is realized by rapid solidification. The three-dimensional metal parts are finally formed by stacking the materials one by one and layer by layer. LDED technology has several significant advantages: First, it can process a variety of metals and alloys, including functional gradient materials, to meet the diversified performance requirements. Second, the process usually does not need to close the forming chamber, which is especially suitable for the manufacture of large components. Third, the technology can also be used for precision repair of locally damaged parts, significantly extending the service life of parts, while reducing resource consumption and environmental impact, so it has broad prospects in industrial applications [63,64,65,66,67].

The application of LDED technology in the field of diamond cutting tool manufacturing has not been widely explored, but its potential is of great significance. Traxel et al. [68] have carried out cutting-edge work in this field. They have successfully fabricated WC-Co/diamond composite tools by using LDED technology. Firstly, the WC-Co substrate and the WC-Co composite doped with diamond powder were systematically optimized. Finally, the laser power was selected as 475 W and two proportions (2.5 wt% and 5 wt%) of diamond powder were added for deposition. The results of optical microscope analysis showed that the interface between diamond and the WC-Co matrix was well bonded in all experimental groups and no macro defects such as large-scale unmelted parts, perforation, or cracking were observed. This preliminarily proves the feasibility of using the LDED process to prepare such composite tools. Moreover, due to the layered printing principle of laser, a number of enhanced phases are formed during solidification and reheating, which ultimately minimizes the total volume of the build-up edge on the tool surface when cutting aluminum and titanium alloys. Specifically, after 10 min of processing aluminum with WC-Co+5dd alloy tools, the size of the build-up edge is 35% smaller than that of commercially available tools, while when processing titanium with WC-Co+2.5dd alloy tools, the total size of the build-up edge is reduced by 64%.

## 3. Additively Manufactured Resin-Bonded Diamond Tools

Resin-bonded diamond tools have high elasticity, impact resistance, good self-sharpening, and high grinding efficiency and are favored by the machining industry because of their excellent machinability [69]. How to improve the abrasion resistance of resin-bonded abrasives and prolong the service life of abrasives is a continuous research topic in the field of grinding and polishing, which has important economic value [70]. The traditional preparation methods of resin-bonded diamond tools, such as the hot-press molding process, have the following core processes: raw material mixing, mold preparation, hot press molding, curing treatment, demolding, and post-treatment. The process is cumbersome and the production cycle is relatively long, which makes it difficult to realize rapid prototyping of complex structural parts [71]. Therefore, the additively manufactured method is introduced to prepare resin-bonded diamond tools with a complex structure, excellent heat dissipation performance, high wear resistance, and long life. Nowadays, the additive technologies used to manufacture resin-bonded diamond tools mainly include selective laser sintering (SLS), stereolithography (SLA), and 3D gel printing (3DGP).

### 3.1. Fabrication of Resin-Bonded Diamond Tools by SLS

**Figure 10:**
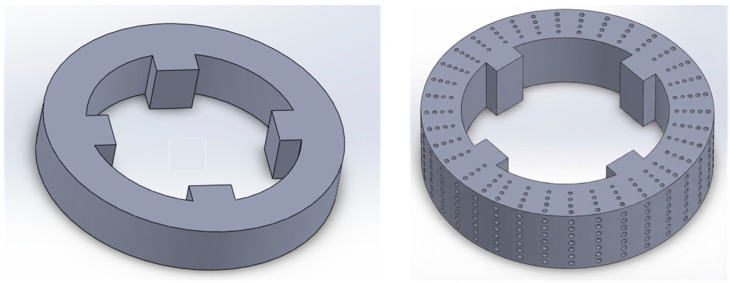
Resin-bonded diamond grinding wheel model with internal cooling holes.

SLS technology is not only widely used in the manufacture of metal-bonded diamond tools but also shows significant potential in the preparation of resin-bonded diamond tools. Chen et al. [72] successfully fabricated a resin-bonded diamond grinding wheel by using SLS technology and designed and manufactured an internal flow channel microstructure in its working layer. In this study, the grinding test of typical hard and brittle materials, such as glass, alumina ceramics, and cemented carbides, was carried out by using this 3D-printed grinding wheel. The experimental results show that the resin-bonded diamond grinding wheel fabricated by SLS technology can effectively realize the precision grinding of these hard and brittle materials. Its unique internal flow channel microstructure plays a key role: it significantly enhances the cooling efficiency in the grinding process, and helps to reduce the temperature of the grinding area, thus reducing the grinding force, while improving the processing quality of the workpiece surface and achieving lower surface roughness. This research introduces a novel approach for the fabrication and performance optimization of resin-bonded grinding wheels with complex structures. Du et al. [73] successfully fabricated a resin-bonded diamond grinding wheel with an internal flow channel microstructure by using SLS technology (see Figure 10 for the structure diagram). In this study, nylon was selected as the resin bond, and diamond abrasive particles, hollow glass beads, and white corundum were added as composite fillers. The mechanical properties and microstructure of the grinding wheel were systematically analyzed. The results show that the SLS process can effectively realize the strong bonding of diamond abrasive particles in the resin matrix. The addition of white corundum significantly improved the hardness and bending strength of the grinding wheel, but a slight increase in grinding force was also observed in the process of grinding glass. In addition, increasing the coolant supply in the grinding arc area can effectively reduce the friction between the abrasive and the workpiece, improve the heat dissipation conditions, and improve the grinding performance. The results verify the feasibility of preparing a resin diamond grinding wheel with a complex internal flow channel microstructure based on SLS technology and provide a new scheme for the manufacture of high-performance customized grinding wheels.

### 3.2. Fabrication of Resin-Bonded Diamond Tools by SLA

**Figure 11:**
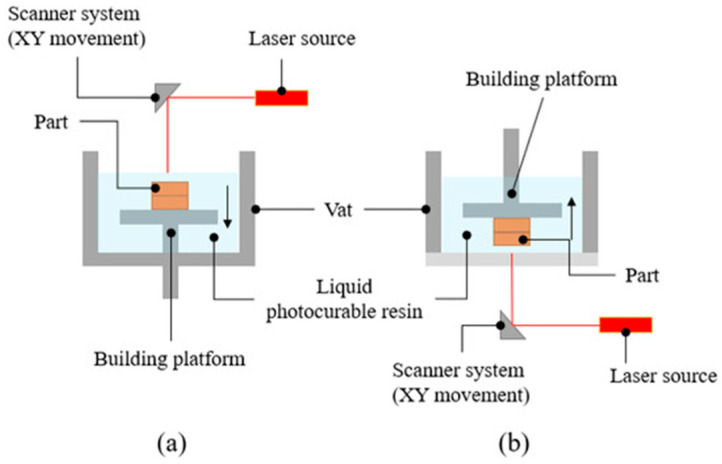
Basic principle of SLA: (**a**) top-down; (**b**) bottom-up. Adopted with permission from Bove et al. [76].

SLA is one of the earliest developed additively manufactured technologies. The process is shown in Figure 11. According to the different construction methods, it can be divided into bottom-up and top-down. The liquid resin is selectively cured through photopolymerization to produce three-dimensional objects. When a specific wavelength of light irradiates the resin, the short molecular chain will polymerize, converting the monomer and oligomer into a rigid or flexible curing geometry. In the SLA process, an ultraviolet laser (typically a solid-state or HeCd laser, wavelength 325–405 nm, power 50–500 mW) is used to selectively cure the resin layer by layer according to the sliced 3D model data. Its basic process is similar to other additive manufacturing methods. First, STL files are generated by computer modeling and then imported into the printer software. In the software, the object will be sliced layer by layer (sliced on the XY plane) and constructed layer by layer in the Z direction. Stereolithography technology has the advantages of a high molding speed, high degree of automation, and high dimensional accuracy and can manufacture objects with a complex internal geometry [74,75,76].

Research on UV-curable resin-bonded abrasives has confirmed that they have significant advantages over traditional abrasives. The abrasive performance of these abrasives largely depends on the characteristics of the mixed resin. Huang et al. [77] systematically discussed the effects of the UV irradiation time and diamond concentration on the properties of abrasive resin mixed specimens. They used UV curing technology to prepare a diamond grinding wheel based on resin 425. The research found that the elongation of mixed resin was a key factor affecting the performance of the grinding wheel. The curing depth of the resin decreases with the increase in diamond concentration, but increases with the extension of the UV irradiation time. When the curing time is 80 s, the average surface roughness of the processed workpiece reaches the optimal value. On the other hand, Yang et al. [78] studied the effect of surface modification of diamond powder on its bonding with UV-curable resin matrix. They successfully fabricated Al_2_O_3_ coating on the surface of diamond powder. The research results show that the coating can not only effectively improve the oxidation resistance of diamond powder but also reduce its UV absorbance. This modification significantly improved the interfacial bonding between diamond powder and the resin matrix, thereby enhancing the overall mechanical properties of the composite material.

Aiming at the problem that the abrasive particles prepared by the traditional method are disorderly distributed, which leads to an uneven force of the abrasive particles during the machining process and leaves them easy to fall off, as early as 2001, Tanaka et al. [79] fabricated polishing discs and grinding wheels that can tightly adsorb and evenly distribute abrasive grains by UV curing technology. Qiu et al. [80,81,82] used light curing technology to prepare resin-bonded diamond wheels with a three-dimensional controllable abrasive grain arrangement. The early research mainly focused on two kinds of light-cured resin diamond (without Al_2_O_3_ and containing 15 wt% Al_2_O_3_ resin). On the one hand, the influence of the diamond abrasive exposure height on the holding force was analyzed; on the other hand, the effect of the Al_2_O_3_ content on tensile strength was discussed. The results show that the resin binder with micron alumina particles can enhance the holding force of the resin on diamond under the same conditions of exposed abrasive particles. The higher the exposure height, the lower the holding power of the resin. When the mass percentage is 15%, the effect is the most significant. The ready-made light curing equipment cannot directly manufacture this grinding wheel, so a set of devices for manufacturing a resin-bonded diamond grinding wheel with a three-dimensional controllable abrasive arrangement is designed. Light-cured resin diamond wheels with different abrasive space configurations were fabricated, and confirmatory grinding tests were carried out. The experimental results show that under the same process conditions, the grinding wheel with orderly arranged abrasive grains has a longer effective grinding time and greater total material removal than the wheel with radially arranged abrasive grains. In the grinding process, the smaller the change in the number of abrasive particles in contact with the workpiece surface, the higher the stability of the grinding process.

**Figure 12:**
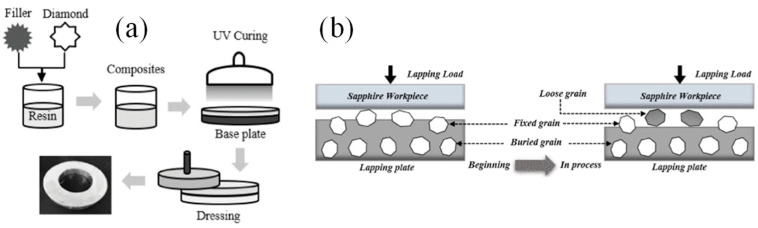
(**a**) The fabrication process of the ultraviolet-curable resin bond diamond lapping plate; (**b**) Schematic diagram of the ultraviolet-curable resin bond lapping plate. Adopted with permission from Guo et al. [84].

Guo et al. [83,84] proposed a new abrasive processing tool fabricated by UV-curable resin and diamond abrasive particles. The process and structure are shown in Figure 12a,b. In order to ensure that the UV energy absorbed in a small area and the corresponding light reaction are uniform, a fan-shaped manufacturing method was used in the curing process and the grinding effect was compared with the traditional iron plate grinding process. The experimental results show that the surface roughness parameters of a UV-curable resin-bonded abrasive plate can be reduced by about 10% compared with those of the iron plate. In the study of evaluating the material removal rate based on the weight loss of the workpiece, the material removed by resin plate grinding in the stable processing state is reduced by about 25% per minute. For the surface finishing of sapphire substrate, the grinding disc manufactured by this technology performs well, and its surface roughness value is 0.22 μm, which is reduced by 45% compared with the traditional fixed abrasive grinding process. Although the slurry-based grinding process can achieve a surface finish of 0.18 μm, the material removal rate is only 3.08 mg/min. In contrast, the material removal rate of the process was increased to 6.19 mg/min by using the grinding disc manufactured by the UV curing technology. It can be seen that the fabricated UV-curable resin tool cannot only achieve a surface quality comparable to the slurry grinding process but also avoid the loss of material removal efficiency.

### 3.3. Fabrication of Resin-Bonded Diamond Tools by 3DGP

**Figure 13:**
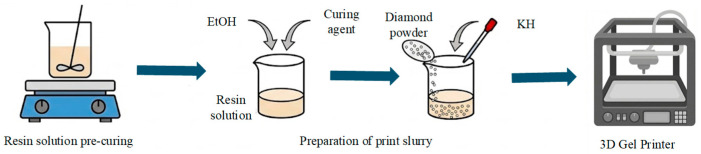
Schematic diagram of 3DGP manufacturing resin-bonded diamond tool.

Ren et al. [85] proposed a new additively manufactured process: 3DGP based on thermoplastic 3D printing (3DTP). This process combines the advantages of direct inkjet printing and the gelation process and has the significant advantages of a wide range of materials, low equipment cost, and high printing efficiency. The 3DGP process mainly includes the following steps: premixed solution preparation, slurry preparation, initiator addition, and printing. Shao et al. [69] successfully fabricated the porous structure of phenolic resin-bonded diamond tools using 3DGP technology. As shown in Figure 13, the preparation process mainly includes four steps: resin pre-curing, printing slurry preparation, 3DGP layer-by-layer printing, and curing molding. The results show that when the ratio of phenolic resin to absolute ethanol is 2:1, the rheological properties of the slurry are most suitable for printing and the molding effect is the best. When the size of the printing needle is 0.60 mm and the parameter combination of layer height and line width is 0.48 mm, the surface quality of the printed part is good. This technology realizes the accurate and controllable design and manufacture of porous diamond tools in terms of pore structure and porosity, and the porous structure shows a good performance, which fully proves the feasibility and application potential of 3DGP technology in the preparation of resin-bonded diamond tools with a complex structure.

## 4. Additively Manufactured Ceramic-Bonded Diamond Tools

Ceramic-bonded diamond tools are widely used in the precision processing of hard and brittle materials such as cemented carbide, monocrystalline silicon, advanced ceramics, and gemstones because of their excellent sharpness, good shape retention, high self-sharpening, long service life, and high processing efficiency [86]. However, the traditional preparation process (mainly atmospheric pressure sintering) has clear limitations, such as a high sintering temperature, long time, and low density, which can easily cause an abnormal growth of grains, resulting in microstructure coarsening and a large fluctuation of mechanical properties [87]. In addition, although the traditional method can form pores, it is difficult to accurately control the size, morphology, and distribution of pores, and the porosity has a key impact on the performance, heat dissipation, and self-sharpening of the diamond tools [88]. In order to break through these limitations, additively manufactured technology has been introduced into this field. Digital light processing (DLP) and direct ink writing (DIW) have become the main additive processes for the preparation of ceramic-bonded diamond tools with complex structures.

### 4.1. Fabrication of Ceramic-Bonded Diamond Tools by DLP

**Figure 14:**
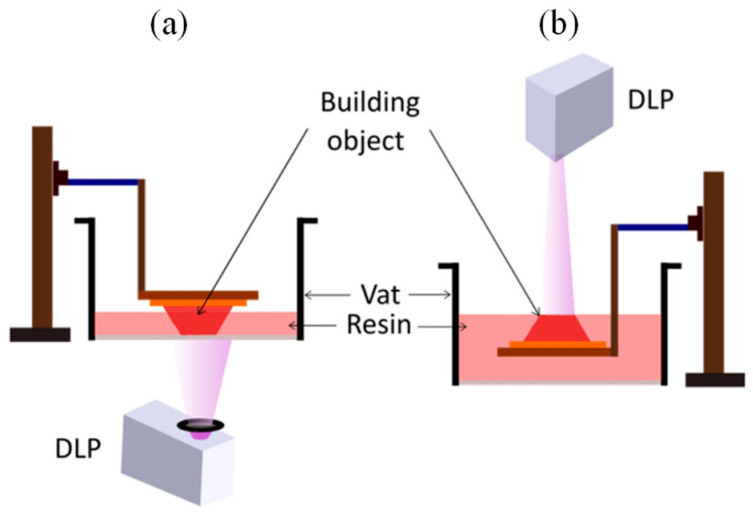
Basic principle of DLP: (**a**) bottom-up; (**b**) top-down. Adopted with permission from Chaudhary et al. [90].

DLP technology is a light-curing, additively manufactured technology based on an ultraviolet light source and digital micro-mirror device (DMD) chip. Its working principle is as follows: the DMD chip digitally modulates the image signal, and the UV projector projects the graphics of each layer onto the slurry surface in the printing tank in the form of surface exposure at one time, so that the whole layer of ceramic suspension can be photopolymerized and cured at the same time. The process repeats layer by layer and finally constructs a three-dimensional entity. There are two construction modes: bottom-up and top-down (see Figure 14). With its surface exposure characteristics, DLP technology achieves a faster printing speed and higher molding efficiency and has more advantages in equipment stability and processing cost. Although DLP technology was primarily developed for pure resin systems at first, recent studies have proved that it shows great potential in the photopolymerization treatment of a ceramic suspension: through the implementation of appropriate degreasing and sintering processes after printing, it can successfully manufacture ceramic parts with a fine structure and excellent performance [89,90,91].

**Figure 15:**
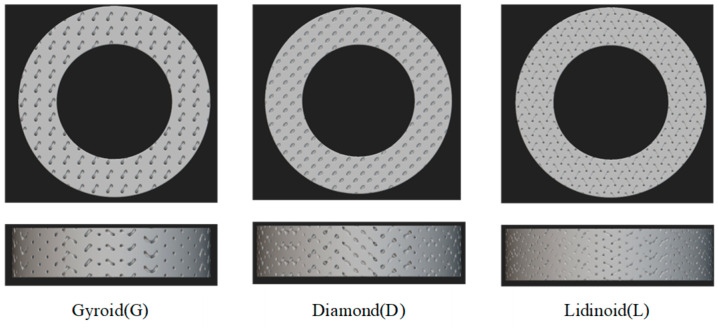
Three grinding wheels with different TPMS structures.

Chen et al. [92,93] used DLP technology to prepare a porous-structure ceramic-bonded diamond grinding wheel based on TPMS. Three grinding wheels with different TPMS structures are shown in Figure 15. By optimizing the formula of the ceramic matrix and diamond slurry, the uniform distribution of diamond abrasive particles and good connectivity of the pore structure were achieved. The results show that the sintering temperature has a significant effect on the microstructure and mechanical properties of the grinding wheel: with the increase in sintering temperature, the porosity gradually decreases and the density increases; however, when the temperature exceeds the optimal value (about 700 °C), the pores in the green body cannot be effectively discharged, resulting in over-burning, foaming, and other phenomena, but increasing the number of pores and reducing the strength of the sample. In addition, when sintered below 700 °C, the diamond abrasive grains can maintain a complete crystal structure. When the temperature reaches 700 °C, some of the diamond abrasive particles begin to transform into amorphous carbon, which weakens the grinding performance of the grinding wheel. It was determined that 680 °C was the optimal sintering temperature, and the grinding wheel fabricated under these conditions showed a good comprehensive grinding performance. Through the grinding test of SiC ceramics, it is found that compared with the traditional solid-structure grinding wheel, the TPMS porous grinding wheel fabricated by DLP has advantages in material removal rate, grinding temperature control, and workpiece surface roughness: the workpiece surface roughnesses after grinding by the four groups of grinding wheels are 0.178 μm, 0.056 μm, 0.128 μm, and 0.343 μm, respectively, indicating that the porous grinding wheel fabricated by DLP technology can obtain a better surface processing quality under similar diamond particle sizes. The results also showed that the compressive and flexural strength of the porous ceramic matrix decreased with the decrease in porosity. When the porosity is 30%, the sintering shrinkage can be controlled within 23%. By increasing the solid content of the slurry, the strength of the grinding wheel can be effectively enhanced and then the surface roughness can be improved. In different TPMS configurations, the G-plane structure grinding wheel has the highest strength, showing the largest material removal rate and grinding ratio; however, the L-plane structure grinding wheel has a large loss in the grinding process due to its low strength.

### 4.2. Fabrication of Ceramic-Bonded Diamond Tools by DIW

**Figure 16:**
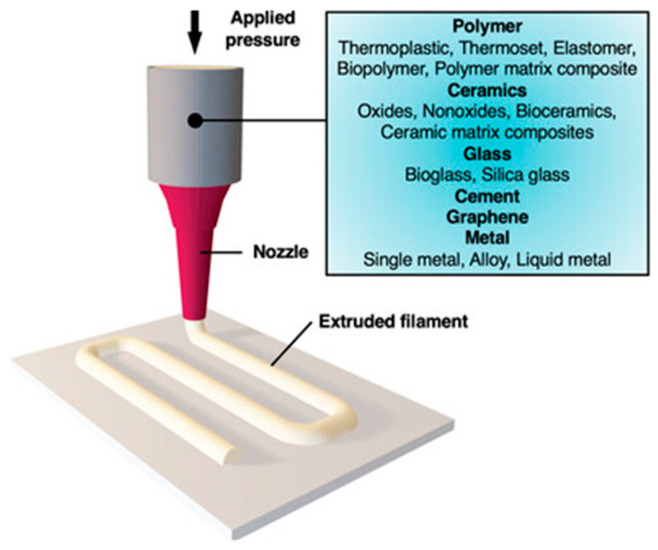
Basic principle of DIW. Adopted with permission from Saadi et al. [96].

DIW is a layer-by-layer printing technology based on extrusion. The printing material used is a non-Newtonian fluid viscous slurry with composite rheological properties at room temperature, which has a specific solid–liquid two-phase structure. The DIW schematic diagram is shown in Figure 16. Generally, the DIW process includes three steps: first, use CAD software to generate 3D modeling of the structure, then obtain the 3D motion path file of the nozzle through the slicing software, and finally, deposit slurry under pressure driven by the fine nozzle. As an advanced additively manufactured method, the core advantage of DIW technology is that it can directly construct hollow objects without a supporting structure, which makes it especially suitable for manufacturing complex and high-precision porous scaffolds. This technology has few restrictions on the types of materials. As long as the slurry used has appropriate rheological properties, it can be accurately printed into a three-dimensional structure with high-resolution patterns, high degrees of freedom, and unique material properties through DIW technology. In addition, DIW technology also has the clear advantages of simplified post-processing steps and less material waste, so that the whole manufacturing process is not only more economical and efficient but also more environmentally sustainable [94,95,96,97,98].

**Figure 17:**
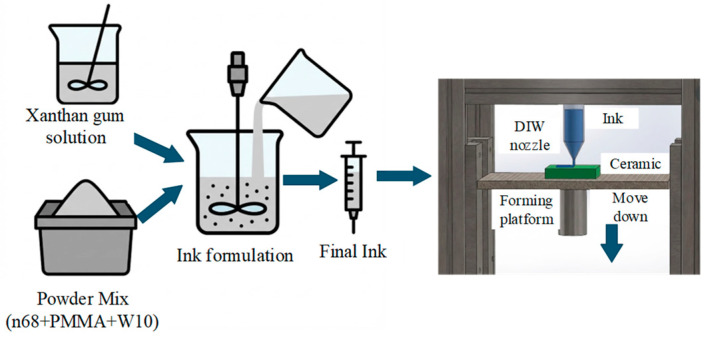
Preparation process of ceramic-bonded diamond grinding wheel.

Lu et al. [99] successfully fabricated a diamond grinding wheel with a honeycomb porous structure by using DIW technology. Using 70–80 wt% ceramic powder and 20–30 wt% diamond powder as mixed raw materials, the tool accurately formed the structural unit with a hexagonal honeycomb through-hole by the DIW process, and the side length of the single hole was controlled in the range of 2–3 mm. This ordered porous structure significantly optimizes the chip holding and chip removal space in the grinding process, which can effectively remove the debris generated by grinding and accelerate the heat dissipation, so as to avoid the thermal damage of abrasive tools caused by chip blockage. At the same time, the structural design is also helpful to maintain the self-sharpening of the abrasive, as well as improve the processing efficiency and tool life. Huang et al. [100] successfully fabricated three types of ceramic-bonded diamond grinding wheels, with a solid structure, triangular structure, and lattice structure, using DIW technology (the process flows are shown in Figure 17). The effect of the xanthan gum (XG) content on the rheological properties of ceramic ink was systematically discussed, and the effects of the sintering temperature and pore former (polymethyl methacrylate, PMMA) content on the key properties of the grinding wheel, such as size shrinkage, microstructure, mechanical strength, and porosity, were analyzed. The experimental results show that when 3 wt% XG solution is added, the ceramic slurry can maintain a good elastic state, which is suitable for DIW molding. With the increase in PMMA content, the open porosity of the grinding wheel initially showed a downward trend, while the closed porosity increased significantly. When the PMMA content reached 30 vol%, the open porosity increased to the maximum. The results show that although PMMA cannot significantly improve the total porosity, it can form regular and uniform spherical pores, which can effectively improve the uniformity and stability of grinding results, while the bending strength of the grinding wheel is not significantly reduced. In terms of grinding performance, the three kinds of grinding wheels can obtain an equivalent surface quality when machining sapphire substrate, but the triangular-structure and lattice-structure grinding wheels show better material removal efficiencies, which are 1.6 times and 1.8 times higher than those of solid-structure grinding wheels, respectively. This study confirmed that the ceramic-bonded diamond grinding wheel fabricated by DIW technology can achieve accurate control of porosity and has excellent self-sharpening and an efficient grinding performance, which provides a reliable way to manufacture a high-performance grinding wheel with a complex structure.

## 5. Future Market Trends of Additive Manufacturing

The diamond tool industry currently stands at a critical strategic juncture. While traditional manufacturing technologies—such as sintering and electroplating—have underpinned decades of market maturity, they are increasingly approaching their asymptotic limits in terms of geometric complexity and functional integration. The demand for next-generation tools, characterized by complex internal cooling channels and optimized porous structures, is exposing the inherent constraints of conventional methods. Consequently, the adoption of additive manufacturing represents not merely a technical expansion but an economic imperative for the industry’s future.

**Figure 18:**
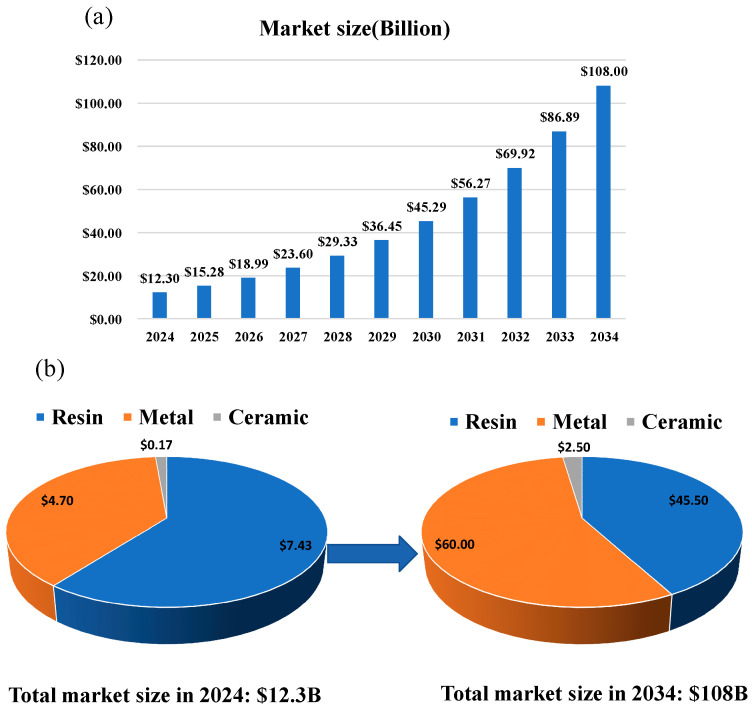
(**a**): Global Additive Manufacturing (AM) Overall Market Forecast (2024–2034); (**b**): AM Market Share Changes from 2024 to 2034 (by material). The data is sourced from VoxelMatters’ 2025 Additive Manufacturing Market Report.

Figure 18 provides crucial quantitative evidence. This chart is based on the latest market report from the authoritative institution VoxelMatters (2025). Figure 18a first presents the macro trend: The global AM market is in a high-speed expansion channel and is expected to grow from 12.3 billion USD in 2024 to 108 billion USD in 2034, with a compound annual growth rate (CAGR) as high as 24.4%. However, Figure 18b reveals a more significant internal structural transformation. In 2024, the market landscape continued to be characterized by its historical roots of “Rapid Prototyping”, with Resin/Polymer occupying a dominant share of 60.4% (USD 7.43 B). In contrast, although the metal sector is important, it still ranks second (38.2%). Looking forward to 2034, the market will undergo a fundamental inversion. The metal sector is expected to surge to 60 billion USD, holding an absolute dominant position of 55.6% in the market. Meanwhile, the share of resin/polymer will shrink to 42.1%.

**Figure 19:**
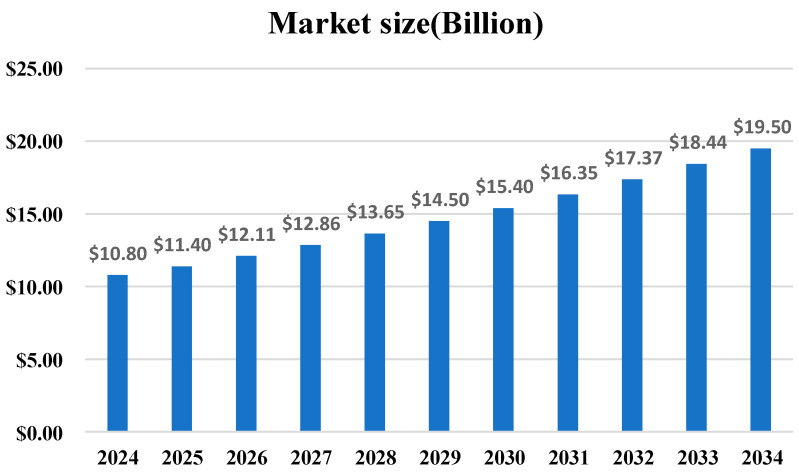
Global Diamond Tools Overall Market Forecast (2024–2034). The data is sourced from Global Market Insights’ Diamond Tools Market Size Report.

The market prospects of the diamond tool industry are extremely broad in the next ten years. According to the latest market size forecast (Figure 19), the industry is expected to grow from 10.8 billion USD in 2024 to 19.5 billion USD in 2034, with a total growth rate exceeding 80%. This continuous linear growth trend indicates that as the demand for precision manufacturing and processing of hard and brittle materials increases, the market value of diamond tools, as key processing consumables, will continue to be realized. The leading enterprises of diamond tools at home and abroad mainly include Saint-Gobain of France, DISCO, and Asahi Diamond of Japan. The domestic companies mainly include Bosun Co., Ltd. (Shijiazhuang, China) and Worldia (Beijing, China).

For traditional manufacturers, “Additive Manufacturing of Diamond Tools” is not merely a new process; it is a lever to break the homogeneity of the mature market. By leveraging AM to manufacture tools with complex cooling channels and optimized porous structures, companies can unlock high-value-added applications that traditional sintering cannot achieve. Understanding the state of the art in AM diamond tools is the prerequisite for researchers and industries to capture this high-growth opportunity in the coming decade.

## 6. Conclusions

This paper systematically reviews the current research status and development trends of additively manufactured technology in the field of metal-bonded, resin-bonded, and ceramic-bonded diamond tool manufacturing. The comprehensive analysis of the existing literature shows that although additively manufactured diamond tools have developed to varying degrees in the design and manufacture of these three types of matrix diamond tools, they are facing a series of key technical bottlenecks and scientific challenges in their respective fields.

Metal-bonded additive manufacturing has made remarkable progress after 20 to 30 years of development, but it also faces the challenges of severe interfacial reaction between metal powder and diamond, serious thermal damage (graphitization) of diamond, and large residual stress. The light curing process is usually used to manufacture diamond tools with resin-bonded additives, but shrinkage deformation can easily occur during the curing process of the photopolymer resin, and the mechanical properties and heat resistance of the matrix are relatively poor. The manufacturing of diamond tools with ceramic-bonded additives is brittle, with poor impact resistance and poor compatibility between ceramic materials and diamond particles, and ceramic materials usually need high-temperature sintering, which increases the production cost.

A comparison across different additive manufacturing methods indicates that metal-bonded processes such as SLM and SLS exhibit a superior tool hardness, thermal resistance, and wear resistance but require the highest equipment investment and energy consumption. Resin-bonded processes including SLA and 3DGP offer simpler equipment and lower energy consumption but result in tools with relatively lower hardness and heat resistance. Ceramic-bonded processes such as DLP and DIW achieve excellent thermal stability and high hardness, although brittleness remains a limiting factor. These distinctions highlight the trade-offs among cost, energy consumption, and functional properties in selecting appropriate AM methods for diamond tool fabrication.

In view of these problems, the following aspects still need continuous attention and research in the future:

(1) Tools Material Research and Development: Develop pre-alloyed powder with high thermal conductivity, high-strength toughness, and good interface compatibility for diamond tool additive manufacturing; develop photosensitive resin with high strength, high heat resistance, and high wear resistance for additive manufacturing diamond tools; and develop a slurry system specifically designed for additive manufacturing of diamond tools, which features a strong impact resistance, good affinity with diamond, and excellent rheological properties. Each matrix material has its own advantages and disadvantages. A new composite material system dedicated to diamond tools can be developed to achieve the best performance in specific application scenarios.

(2) Optimization of Forming Process: In-depth study of the metal sintering mechanism, optimization of the laser scanning strategy, heat treatment, and other process parameters to reduce thermal damage and residual stress; in-depth study of the resin curing mechanism, optimize the scanning strategy and curing parameters, achieve the matching of curing depth and light intensity, reduce resin scorch and particle stratification, and prevent over-curing; and in-depth study of the ceramic sintering mechanism, optimization of the scanning path and energy density matching, and low-temperature sintering route development in order to reduce hot cracks and reduce costs.

(3) Innovative Design of Structure: Optimize the external structure of the tool to realize the innovative design of special-shaped, thin-walled, and complex diamond tools, so as to meet the needs of different industries, such as aerospace and geological exploration, for special-shaped diamond tools; and optimize the internal structure of the tool to achieve an innovative design of structured porous diamond tools featuring an ordered arrangement of abrasive particles, ordered arrangement of pore structures, and ordered arrangement of flow channels, so as to improve the heat dissipation performance, chip holding performance, and self-sharpening performance of the tool.

(4) Integrated Additive and Subtractive Manufacturing of Diamond Tools: Additively manufactured technology can maximize the design freedom of diamond tools, but there are few existing studies on the dressing of additively manufactured diamond tools. The integrated manufacturing technology of additive and subtractive materials can be used to complete the forming and dressing of diamond tools with a complex structure at the same time, which is one of the future development trends.

(5) Development of special printing equipment for diamond tools: The artificial intelligence system is embedded in the equipment, and the key data in the printing process are collected in real time by using high-precision sensors. The printing status is dynamically determined by image recognition and an AI algorithm, and the key parameters, such as laser power, scanning path, and powder laying amount, are automatically adjusted to achieve intelligent closed-loop control, real-time monitoring of defects (such as cracks, pores, and diamond particles falling off), and immediate feedback. The system can make adaptive corrections according to the historical data model, so as to improve the printing success rate and consistency.

## Figures and Tables

**Table 1:** Laser power range and wavelength corresponding to different processes.

Process	Laser Power Range	Wavelength
SLM	100–1000 W	1070 nm
SLS	100–1000 W	1070 nm
FDMS	100–500 W	808–1070 nm
LDED	500–6000 W	808–1070 nm
SLS	20–100 W	10,600 nm
SLA	50–500 mW	325–405 nm
3DGP	10 mW–2 W	355–810 nm
DLP	30–200 W	365–405 nm
DIW	NO	NO

## Data Availability

No new data were created or analyzed in this study. Data sharing is not applicable to this article.
